# Supplementary material for: Reliability and signal comparison of OPM-MEG, fMRI & iEEG in a repeated movie viewing paradigm
Source: Imaging Neurosci (Camb). 2026 May 8;4:IMAG.a.1218. doi: 10.1162/IMAG.a.1218 (PMC13159027; doi:10.1162/IMAG.a.1218)
Supplement: Supplementary Material [file IMAG.a.1218_supp.pdf]

Supplementary Material

Reliability and signal comparison of OPM-MEG, fMRI & iEEG in a repeated movie viewing paradigm

Olivia R. Christiano & Sebastian Michelmann

Table S1: Comparison of within-subject maximum correlations after temporally down-sampling and averaging.

Modality	Frequency		Maxima			r (20 Hz)	r (0.67 Hz)	r (0.67 Hz, GA)
			z	MNI Coord.	AAL Label (Left/Right)			
OPM	δ	0.5–4 Hz	13.42	8, –96, 16	Cuneus (R)	.08	.19	.61
	θ	4–8 Hz	16.04	8, –96, 16	Cuneus (R)	.08	.20	.55
	α	8–12 Hz	13.57	0, –88, 32	Cuneus (L)	.08	.23	.63
	β	12–28 Hz	12.28	–8, –96, 24	Cuneus (L)	.04	.22	.65
	γ_1	28–46 Hz	6.01	–40, –16, 64	Precentral (L)	.02	.12	.42
	γ_2	55–70 Hz	5.85	0, 56, 0	Frontal Sup Medial (L)	.02	.12	.30
	HF	64–116 Hz	5.28	40, 56, 8	Frontal Mid (R)	.02	.11	.43
	BB	0.5–116 Hz	20.76	0, –96, 24	Cuneus (L)	.07	.30	.66
fMRI	–	–	13.75	–56, –16, 0	Temporal Mid (L)	.35	.32	.71
iEEG	δ	0.5–4 Hz	12.35	–58, –14, 48	Postcentral (L)	.29	.58	–
	θ	4–8 Hz	9.79	65, –18, 25	SupraMarginal (R)	.22	.66	–
	α	8–12 Hz	10.75	–68, –11, 8	Temporal Sup (L)	.24	.59	–
	β	12–28 Hz	9.74	–67, –21, 16	SupraMarginal (L)	.11	.74	–
	γ_1	28–46 Hz	7.80	–48, –54, –24	Temporal Inf (L)	.09	.73	–
	γ_2	55–70 Hz	8.30	65, –20, 15	Temporal Sup (R)	.12	.51	–
	HF	64–116 Hz	21.26	–67, –21, 16	SupraMarginal (L)	.34	.86	–
	BB	0.5–116 Hz	12.30	–68, –11, 8	Temporal Sup (L)	.25	.82	–

Note. For each imaging modality and frequency band, we identify the maximum within-subject ***z***-score (surviving multiple comparisons correction) and report correlation coefficients at that location. First, we report the untransformed correlation at the peak ***z***-score location using data sampled at 20 Hz (*r* (20 Hz)), where correlations were computed between the first and second movie viewing for each subject and channel and averaged across subjects. Next, we report the corresponding value from the same location using data that were further down-sampled to 0.67 Hz (*r* (0.67 Hz)). Finally, for OPM and fMRI, we averaged the 0.67 Hz time series across subjects separately for viewing 1 and viewing 2, computed the correlation between the two grand-averaged signals, and report the resulting untransformed coefficient (*r* (0.67, GA)). For fMRI, analyses were conducted on data projected to the OPM source grid for consistency. *r* (0.67, GA) is not applicable to the single-subject iEEG data.

Within-viewing correlation analyses

For within-viewing comparisons, we followed the same procedures described in the main Methods section, except we correlated time series from the same viewing (e.g., subject *i*'s first viewing with subject *j*'s first viewing) rather than between them. Correlations were computed separately for viewing 1 and viewing 2, then averaged prior to generating surrogate distributions and computing *z*-scores.

Table S2: Between-subject reliability statistics for OPM and fMRI within movie viewings.

Modality	Frequency Band		% MC	M_z	SD_z	Maxima		
						z	MNI Coord.	AAL Label (Left/Right)
OPM	δ	0.5–4 Hz	94.8%	5.22	2.06	15.33	–32, 24, –32	Temporal Pole Sup (L)
	θ	4–8 Hz	90.1%	5.87	2.80	16.96	24, –96, 0	Calcarine (R)
	α	8–12 Hz	84.6%	6.88	3.09	15.16	–16, –80, 48	Parietal Sup (L)
	β	12–28 Hz	94.2%	6.45	2.46	14.40	–32, –56, 64	Parietal Sup (L)
	γ_1	28–46 Hz	8.0%	3.27	0.56	5.61	0, 64, 0	Frontal Sup Medial (L)
	γ_2	55–70 Hz	4.8%	3.34	0.51	5.88	16, 48, –24	Frontal Sup Orb (R)
	HF	64–116 Hz	9.0%	3.14	0.43	4.75	16, –48, 24	Precuneus (R)
	BB	0.5–116 Hz	99.6%	9.99	3.96	21.46	–24, –96, 0	Occipital Mid (L)
fMRI	–	–	26.6%	4.63	1.99	12.17	–48, –24, 8	Temporal Sup (L)

Note. All statistics are reported for data surviving correction for MC. For each modality and frequency band, this table reports the percentage of voxels/channels/electrodes surviving FDR correction (% MC), the *M* and *SD* of *z*-scores, and the peak *z*-score with its MNI coordinate and anatomical label.

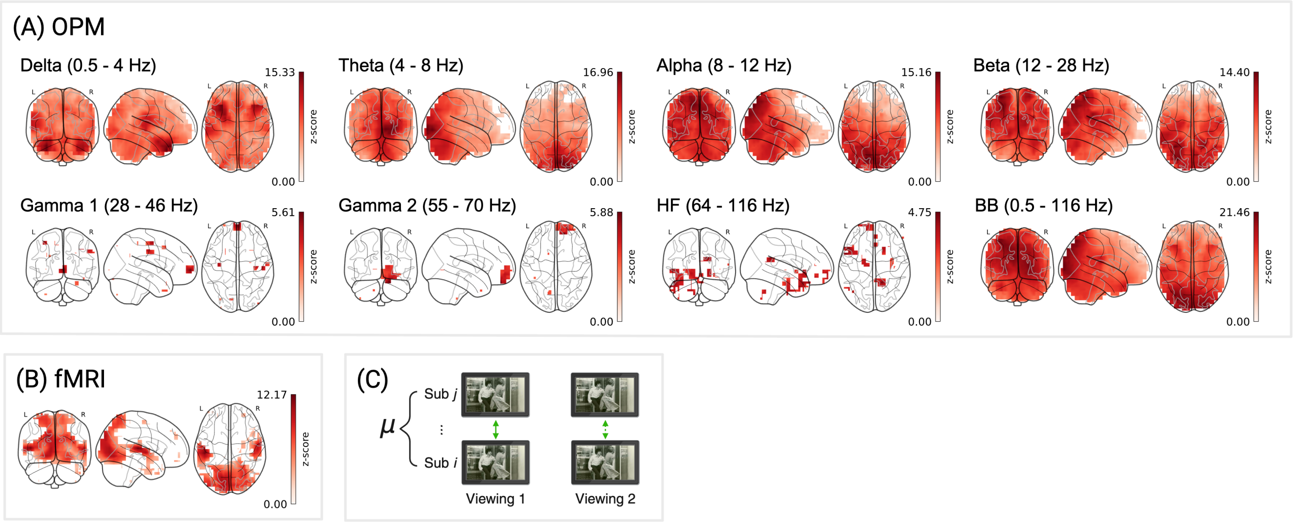

Figure S1: Between-subject reliability for OPM and fMRI within movie viewings. FDR-corrected *z*-scores are shown for: (A) OPM, 3,581 source locations across 10 subjects for 8 frequency bands; (B) fMRI: 3,581 voxels, across 11 subjects. (C) Schematic of between-subject correlation analysis. Color bars reflect *z*-scores (surviving correction for MC).

Table S3: Between-method reliability statistics across frequency bands for OPM, fMRI, and iEEG within movie viewings.

						Maxima		
Comparison	Frequency Band		% MC	M_z	SD_z	z	MNI Coord.	AAL Label (Left/Right)
OPM to fMRI	δ	0.5–4 Hz	4.8%	−0.83	3.83	−7.42	–32, –56, 56	Parietal Sup (L)
	θ	4–8 Hz	11.7%	−2.25	3.71	−11.46	–24, –56, 72	Parietal Sup (L)
	α	8–12 Hz	16.2%	−4.40	3.12	−13.26	–40, –88, 8	Occipital Mid (L)
	β	12–28 Hz	16.1%	−4.22	2.91	−12.03	–24, –88, 24	Occipital Sup (L)
	γ_1	28–46 Hz	0.0%	3.85	0.00	3.85	–24, –8, 64	Frontal Sup (L)
	γ_2	55–70 Hz	1.0%	3.17	2.58	5.74	24, –88, 0	Calcarine (R)
	HF	64–116 Hz	1.1%	3.41	2.64	6.61	0, –96, 24	Cuneus (L)
	BB	0.5–116 Hz	13.7%	−3.88	3.55	−11.69	–16, –56, 64	Precuneus (L)
OPM to iEEG	δ	0.5–4 Hz	3.5%	3.41	1.58	5.45	–21, –55, 78	Parietal Sup (L)
	θ	4–8 Hz	7.3%	3.13	2.35	7.29	–21, –55, 78	Parietal Sup (L)
	α	8–12 Hz	12.2%	3.83	1.58	8.00	–35, –91, 22	Occipital Mid (L)
	β	12–28 Hz	8.1%	3.63	1.28	6.91	–24, –87, 33	Occipital Sup (L)
	γ_1	28–46 Hz	0.0%	–	–	–	–	–
	γ_2	55–70 Hz	0.0%	–	–	–	–	–
	HF	64–116 Hz	0.0%	–	–	–	–	–
	BB	0.5–116 Hz	24.2%	3.67	1.66	8.41	–21, –55, 78	Parietal Sup (L)
fMRI to iEEG	δ	0.5–4 Hz	4.6%	−3.44	2.02	−5.43	–46, –83, 7	Occipital Mid (L)
	θ	4–8 Hz	8.1%	−3.83	1.74	−7.16	–74, –28, 3	Temporal Mid (L)
	α	8–12 Hz	5.3%	−4.74	1.27	−7.41	–67, –8, 3	Temporal Sup (L)
	β	12–28 Hz	5.3%	−4.01	0.90	−6.73	–24, –87, 33	Occipital Sup (L)
	γ_1	28–46 Hz	2.3%	4.28	2.35	6.67	–39, –90, 10	Occipital Mid (L)
	γ_2	55–70 Hz	4.0%	3.97	2.56	7.26	–39, –90, 10	Occipital Mid (L)
	HF	64–116 Hz	6.7%	4.50	1.88	8.08	–24, –87, 33	Occipital Sup (L)
	BB	0.5–116 Hz	8.4%	−4.22	1.07	−6.75	–74, –28, 3	Temporal Mid (L)

Note. All statistics are reported for data surviving correction for MC. For each comparison and band, we report the percent of voxels/electrodes surviving correction for MC (% MC), the *M* and *SD* of *z*-scores, and the peak *z*-score with its MNI coordinate and anatomical label.

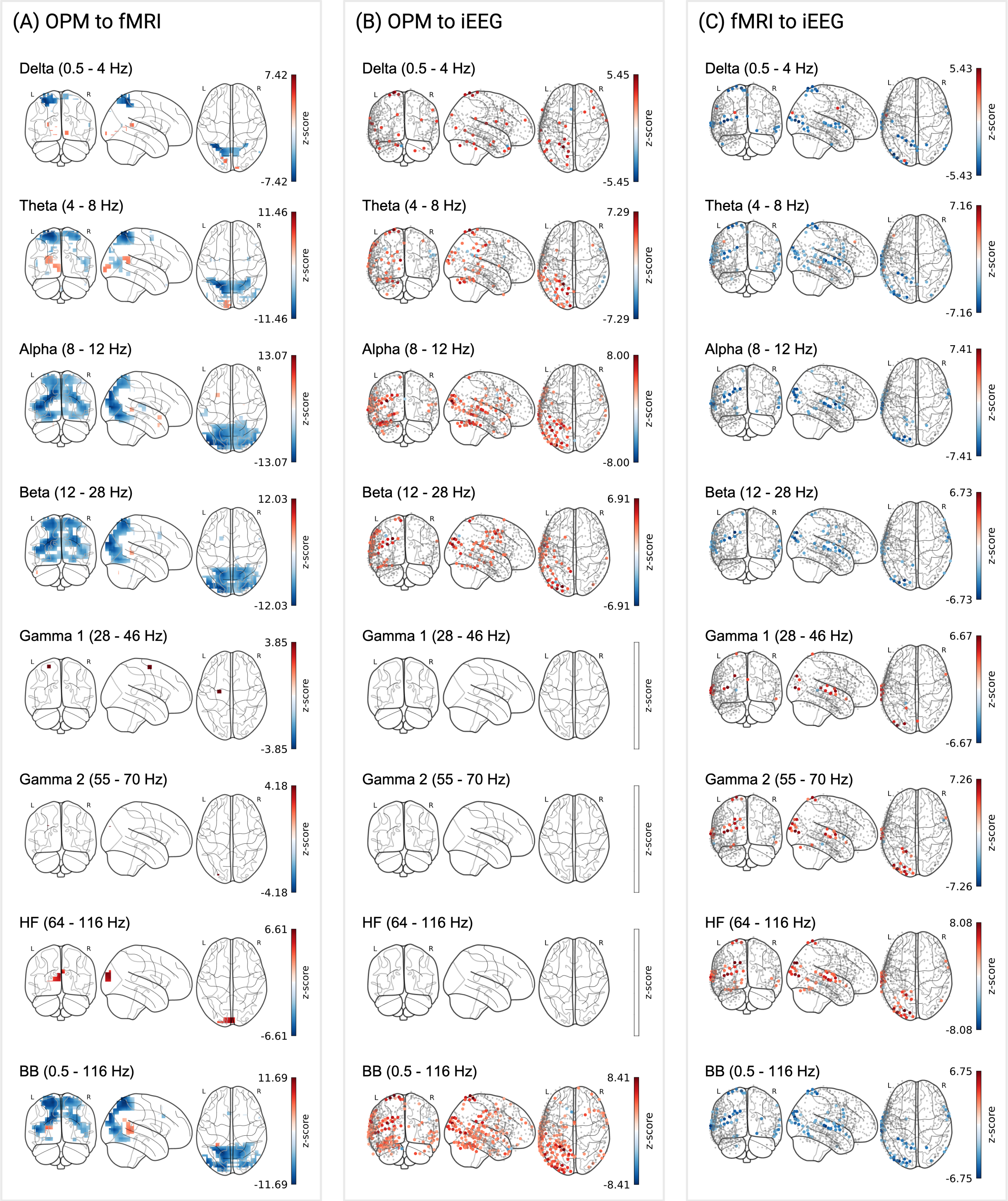

Figure S2: Consistent between-method alignment within movie viewings. (A) OPM to fMRI: *z*-scores from OPM subjects (n = 10) and fMRI subjects (n = 11) are shown for 3,581 locations. (B) OPM to iEEG: *z*-scores from OPM subjects (n = 10) and single-subject iEEG from 656 electrodes pooled across five subjects. (C) fMRI to iEEG: *z*-scores from fMRI subjects (n = 11) and single-subject iEEG from 656 electrodes pooled across five subjects. Color bars reflect *z*-scores (see above).

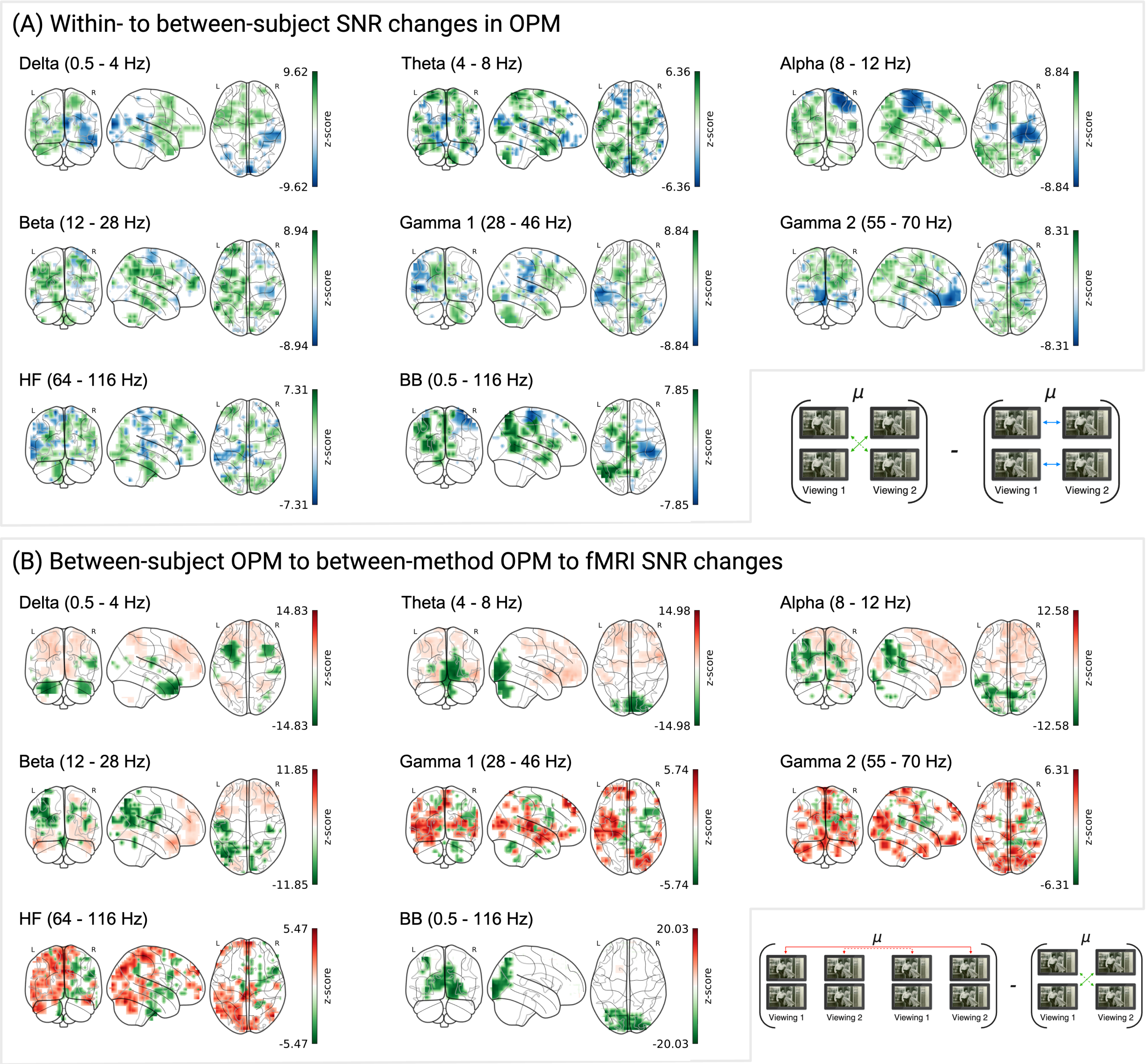

Figure S3: Uncorrected cluster maps of SNR differences within and across modalities. All clusters shown exceed the 97.5th or fall below the 2.5th percentile of the empirical SNR distribution; values were not corrected for multiple comparisons and do not reflect statistical significance. (A) Within-modality SNR changes in OPM, computed by subtracting within-subject from between-subject *z*-scores at each channel. Negative values (blue) indicate greater signal consistency within individuals than across individuals. (B) Between-modality SNR changes comparing between-subject OPM to between-method fMRI, where negative values (red) indicate greater signal reliability within OPM than in cross-modal correspondence. SNR changes are shown across frequency bands from delta (0.5- 4 Hz) to high-frequency (64-116 Hz). Diagrams below illustrate the comparison logic for each panel.

Supplemental analyses with alternate source reconstruction parameters

To ensure that the reliability patterns we observed for OPM were not dependent on the specific source reconstruction parameters used in our main analyses, we repeated all OPM analyses using an alternative inverse solution. Specifically, while our primary results were obtained using LCMV beamforming, we additionally employed a minimum-norm-estimation (MNE) approach, which does not consider the data covariance.

Across source reconstruction approaches, OPM exhibited broadly consistent reliability patterns. Using LCMV beamforming (Table 1, OPM; Table 2, OPM), within- and between-subject reliability was most pronounced in the broadband and lower frequency bands, with effects concentrated in visual and temporal processing regions, while higher-frequency bands showed sparser and more spatially restricted patterns. In our supplemental analyses using an MNE solution (Table S4) reliability estimates were slightly reduced in magnitude overall, and demonstrated less focal spatial distributions, consistent with the smoother nature of minimum-norm estimates (see Figure S4). Nevertheless, similar patterns were observed across source reconstruction methods, indicating that the observed reliability patterns are robust to source reconstruction choices.

Table S4: Within and between-subject reliability statistics for OPM with alternate source reconstruction parameters.

						Maxima	
Modality	Frequency Band	% MC	M_z	SD_z	z	MNI Coord.	AAL Label (Left/Right)
within-subject	δ 0.5–4 Hz	67.7%	3.62	1.32	10.84	48, –16, 40	Postcentral (R)
	θ 4–8 Hz	84.1%	3.57	1.19	8.11	8, –96, 0	Calcarine (R)
	α 8–12 Hz	91.4%	5.24	1.97	9.80	0, 8, 40	Cingulum Mid (L)
	β 12–28 Hz	81.8%	3.83	1.35	8.25	–56, –24, 0	Temporal Mid (L)
	γ_1 28–46 Hz	30.8%	3.02	0.70	5.66	–48, –8, 32	Postcentral (L)
	γ_2 55–70 Hz	28.7%	2.88	0.55	5.31	64, –40, –24	Temporal Inf (R)
	HF 64–116 Hz	63.1%	2.99	0.71	6.76	–56, –48, 48	Parietal Inf (L)
	BB 0.5–116 Hz	99.1%	4.78	1.57	10.11	48, –40, 56	Parietal Inf (R)
between-subject	δ 0.5–4 Hz	54.9%	4.00	1.45	8.76	–64, –16, 32	Postcentral (L)
	θ 4–8 Hz	88.8%	4.03	1.39	9.73	16, –88, –32	Crus II (R)
	α 8–12 Hz	60.5%	3.75	1.34	8.36	–8, –72, 64	Precuneus (L)
	β 12–28 Hz	99.8%	5.21	1.42	9.75	–40, –64, –16	Fusiform (L)
	γ_1 28–46 Hz	15.6%	2.99	0.45	4.50	56, –16, 48	Postcentral (R)
	γ_2 55–70 Hz	15.3%	3.20	0.61	5.40	56, –48, –8	Temporal Mid (R)
	HF 64–116 Hz	45.4%	3.01	0.62	5.50	16, 8, 8	Caudate (R)
	BB 0.5–116 Hz	97.5%	5.36	1.71	12.65	–8, –72, 64	Precuneus (L)

Note. All statistics are reported for data surviving correction for MC. For each modality and frequency band, this table reports the percentage of voxels/channels/electrodes surviving FDR correction (% MC), the *M* and *SD* of *z*-scores, and the peak *z*-score with its MNI coordinate and anatomical label.

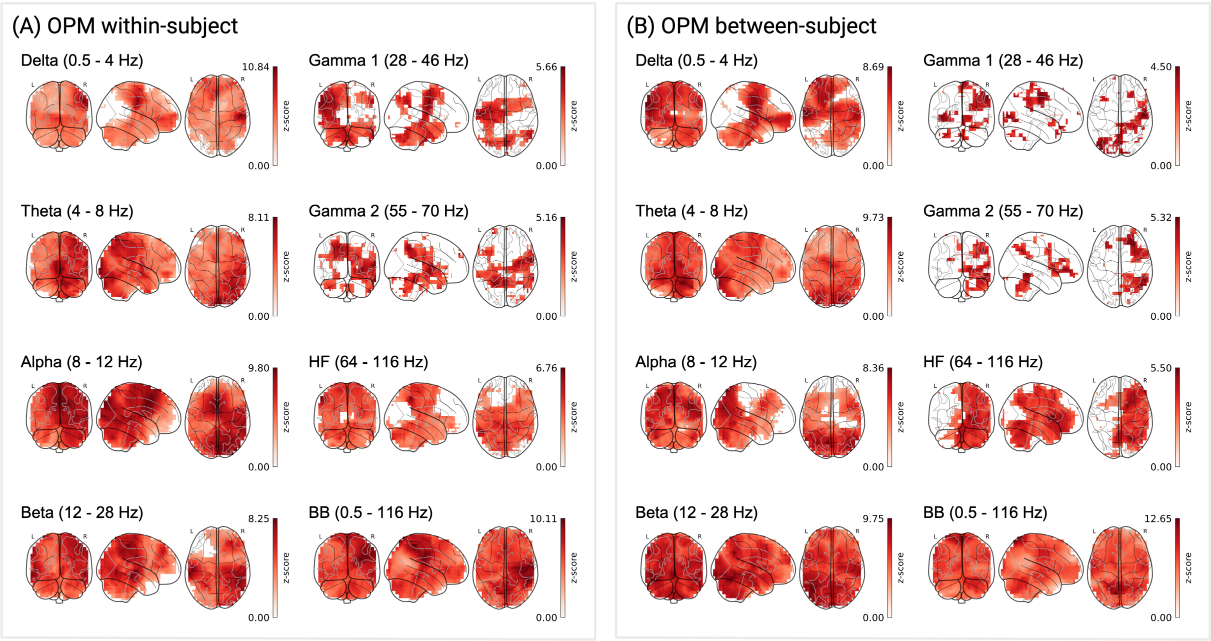

Figure S4: Within and between-subject reliability for OPM with alternate source reconstruction parameters. FDR-corrected *z*-scores are shown for OPM, computed at 3,581 source locations across 10 subjects for 8 frequency bands: (A) within-subject and (B) between-subject comparisons between the first and second movie viewing. Color bars reflect *z*-scores (surviving correction for MC).

A similar pattern was observed for the between-method comparisons (Table 3). Cross-modal reliability between OPM and fMRI, and between OPM and iEEG, using the alternate, MNE reconstruction (Table S5), moderately reduced *z*-score magnitudes, but sometimes increased their spatial extent (see Figure S5). Still, the observed patterns were maintained across both inverse solutions. Together, these findings indicate that the observed patterns of reliability in OPM reflect robust, stimulus-locked structure rather than artifacts of source reconstruction choices, though spatial interpretability may be reduced.

Table S5: Between-method reliability statistics for OPM, fMRI, and iEEG with alternate source reconstruction parameters.

					Maxima		
Comparison	Frequency Band	% MC	M_z	SD_z	z	MNI Coord.	AAL Label (Left/Right)
OPM to fMRI	δ 0.5–4 Hz	0.6%	−3.88	1.68	−5.01	24, –8, 64	Frontal Sup (R)
	θ 4–8 Hz	11.4%	−2.80	3.32	−8.23	–16, –64, 64	Parietal Sup (L)
	α 8–12 Hz	9.3%	−3.79	2.19	−8.95	48, –80, 0	Occipital Mid (R)
	β 12–28 Hz	12.1%	−3.40	2.95	−9.31	–40, –88, 8	Occipital Mid (L)
	γ_1 28–46 Hz	–	–	–	–	–	–
	γ_2 55–70 Hz	2.1%	3.78	0.97	5.51	16, –88, 24	Cuneus (R)
	HF 64–116 Hz	3.6%	4.00	1.19	6.69	–8, –80, 32	Cuneus (L)
	BB 0.5–116 Hz	8.5%	−3.46	2.28	−7.10	–16, –64, 64	Parietal Sup (L)
OPM to iEEG	δ 0.5–4 Hz	1.7%	3.47	0.21	3.91	30, 61, –16	Frontal Mid Orb (R)
	θ 4–8 Hz	7.5%	3.43	1.57	5.66	–29, –49, 73	Parietal Sup (L)
	α 8–12 Hz	3.4%	3.08	2.19	4.88	–35, –91, 22	Occipital Mid (L)
	β 12–28 Hz	4.4%	3.59	1.46	5.44	–8, –56, 70	Precuneus (L)
	γ_1 28–46 Hz	0.2%	4.11	0.00	4.11	–65, 15, 5	Frontal Inf Oper (L)
	γ_2 55–70 Hz	–	–	–	–	–	–
	HF 64–116 Hz	–	–	–	–	–	–
	BB 0.5–116 Hz	17.1%	3.42	0.95	5.84	–58, –22, 47	Parietal Inf (L)

Note. All statistics are reported for data surviving correction for MC. For each comparison and band, we report the percent of voxels/electrodes surviving correction for MC (% MC), *M* and *SD* of *z*-scores, and the peak *z*-score with its MNI coordinate and anatomical label.

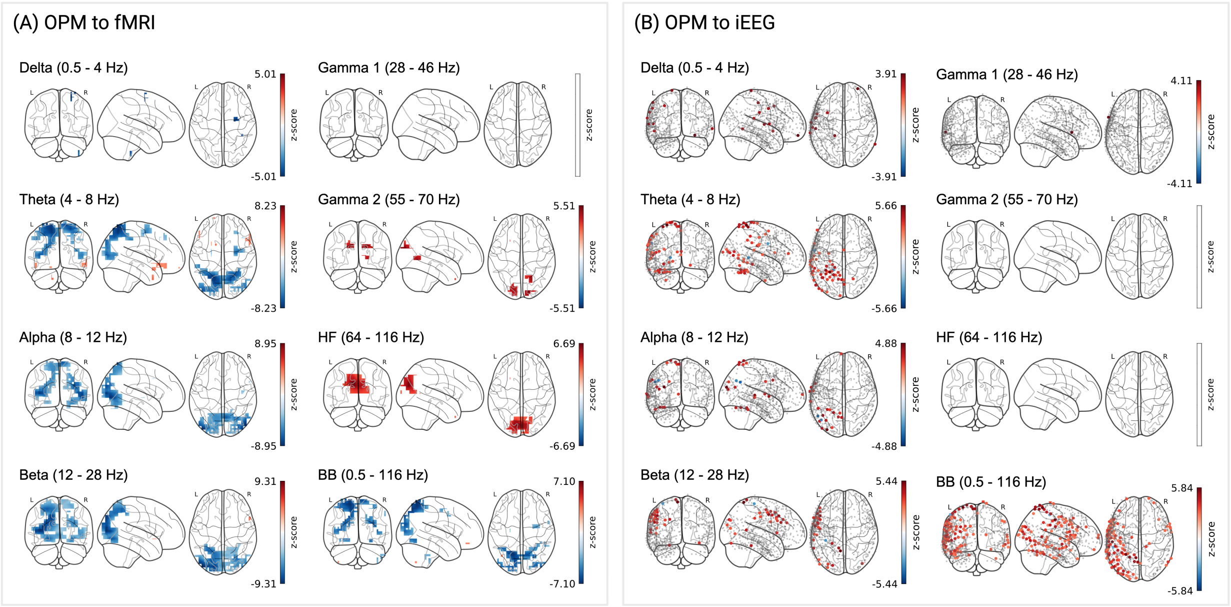

Figure S5: Consistent between-method alignment with alternate source reconstruction parameters. (A) OPM to fMRI: *z*-scores from OPM subjects (n = 10) and fMRI subjects (n = 11) are shown for 3,581 locations. (B) OPM to iEEG: *z*-scores from OPM subjects (n = 10) and single-subject iEEG from 656 electrodes pooled across five subjects. Color bars reflect *z*-scores (see above).

Modality-specific vs cross-modal specific patterns of reliability

In addition to quantifying shared signal across modalities, we identified patterns of reliability exclusive to individual modalities, and regions that emerged as reliable only in cross-modal comparisons. Modality-specific patterns index the distinct sensitivities of each method, including potential differences in depth sensitivity, spatial specificity, and temporal precision. In contrast, cross-modal-specific patterns may reveal stimulus-driven signal that becomes detectable only when modality-specific noise is attenuated (i.e., there is less shared noise between modalities than within them). Examining these patterns therefore clarifies both what is uniquely captured by each modality and what may be missed when relying on any single methodological approach.

To address this, we performed overlap analyses of cross-modal comparisons for OPM to fMRI, and OPM to iEEG, that separated channels into three mutually exclusive categories: (i) channels showing reliable signal within one modality but not the other (e.g., significant *z*-scores in OPM that were not significant in fMRI), (ii) channels showing reliable signal within the second modality but not the first (e.g., significant *z*-scores in fMRI that were not significant in OPM), and (iii) channels that emerged as reliable only in between-method comparisons (e.g., OPM-fMRI) despite not surviving correction within either modality alone. To allow for direct comparison of iEEG within-subject reliability to OPM, we recomputed within-subject analyses using the OPM data that was reconstructed at the 656 iEEG electrode locations from cross-modal analyses. Binary reliability masks of *z*-scores that survived MC correction were created across each comparison and modality, combined to isolate modality-specific and cross-modal-specific channels, and projected into MNI space for visualization. We compared within-subject reliability to between-method reliability across modality pairs, and repeated the same analysis for the between-subject OPM-fMRI comparison.

Table S6: Patterns of reliability exclusive to within-subject OPM, within-subject fMRI, and between-method OPM-fMRI.

						Maxima	
Comparison	Frequency Band	% MC	M_z	SD_z	z	MNI Coord.	AAL Label (Left/Right)
OPM	δ 0.5–4 Hz	33.6%	4.38	1.76	11.01	48, –16, 24	Rolandic Oper (R)
	θ 4–8 Hz	30.7%	4.10	1.97	13.21	8, –88, –16	Cerebellum 6 (R)
	α 8–12 Hz	33.1%	4.45	1.66	9.41	8, –96, –16	Cerebellum Crus II (L)[a]
	β 12–28 Hz	31.3%	3.69	1.42	9.22	16, –80, –24	Cerebellum Crus I (R)
	γ_1 28–46 Hz	6.5%	3.13	0.67	6.01	–40, –16, 64	Precentral (L)
	γ_2 55–70 Hz	5.1%	3.43	0.69	5.85	0, 56, 0	Frontal Sup Medial (L)
	HF 64–116 Hz	13.5%	2.95	0.60	5.09	8, 24, –16	Rectus (R)
	BB 0.5–116 Hz	40.2%	6.28	2.26	13.84	–16, –64, 32	Precuneus (L)
fMRI	– –	0.3%	2.33	0.31	2.86	–16, 24, 56	Frontal Sup (L)
OPM to fMRI	δ 0.5–4 Hz	0.5%	3.69	0.49	4.66	–24, 40, 40	Frontal Sup (L)
	θ 4–8 Hz	0.2%	3.35	0.53	4.40	16, –8, 64	Frontal Sup (R)
	α 8–12 Hz	0.1%	2.98	0.31	3.26	0, –8, –24	Putamen (R) [b]
	β 12–28 Hz	0.8%	3.16	0.33	3.83	16, 0, –8	Pallidum (R)
	γ_1 28–46 Hz	–	–	–	–	–	–
	γ_2 55–70 Hz	–	–	–	–	–	–
	HF 64–116 Hz	–	–	–	–	–	–
	BB 0.5–116 Hz	0.1%	3.22	0.60	3.90	–16, 24, 40	Frontal Sup (L)

Note. All statistics are reported for data surviving correction for MC either in within-subject OPM but not fMRI (OPM, top panel), within-subject fMRI but not OPM (fMRI, middle panel), and between-method OPM-fMRI, but not within-subject OPM nor fMRI (OPM to fMRI, bottom panel). For each comparison and band, we report the percent of voxels surviving correction for MC (% MC), the *M* and *SD* of *z*-scores, and the peak *z*-score with its MNI coordinate and anatomical label.

Table S7: Patterns of reliability exclusive to within-subject OPM, within-subject iEEG, and between-method OPM–iEEG.

					Maxima		
Comparison	Frequency Band	% MC	M_z	SD_z	z	MNI Coord.	AAL Label (Left/Right)
OPM	δ 0.5–4 Hz	50.8%	4.88	2.04	11.21	–7, –99, 16	Cuneus (L)
	θ 4–8 Hz	40.4%	3.83	2.02	13.66	2, –81, 26	Cuneus (L)
	α 8–12 Hz	61.9%	4.76	1.86	12.01	–7, –99, 16	Cuneus (L)
	β 12–28 Hz	56.7%	4.07	1.73	9.88	–21, –101, 15	Occipital Sup (L)
	γ_1 28–46 Hz	11.0%	3.31	0.68	5.23	27, –24, 79	Precentral (R)
	γ_2 55–70 Hz	3.4%	3.67	0.53	4.74	56, 9, –3	Temporal Pole Sup (R)
	HF 64–116 Hz	8.5%	3.10	0.55	4.67	51, 32, 13	Frontal Inf Tri (R)
	BB 0.5–116 Hz	53.2%	6.37	2.83	19.89	–3, –91, 32	Cuneus (L)
iEEG	δ 0.5–4 Hz	–	–	–	–	–	–
	θ 4–8 Hz	–	–	–	–	–	–
	α 8–12 Hz	–	–	–	–	–	–
	β 12–28 Hz	–	–	–	–	–	–
	γ_1 28–46 Hz	–	–	–	–	–	–
	γ_2 55–70 Hz	–	–	–	–	–	–
	HF 64–116 Hz	–	–	–	–	–	–
	BB 0.5–116 Hz	–	–	–	–	–	–
OPM to iEEG	δ 0.5–4 Hz	0.6%	3.44	0.46	4.12	–61, 9, 29	Precentral (L)
	θ 4–8 Hz	0.9%	3.22	0.26	3.58	1, –8, 55	Supp Motor Area (R)
	α 8–12 Hz	0.3%	2.96	0.05	3.00	–35, 65, 10	Frontal Sup (L)
	β 12–28 Hz	0.9%	3.16	0.29	3.52	–65, –55, 1	Temporal Mid (L)
	γ_1 28–46 Hz	–	–	–	–	–	–
	γ_2 55–70 Hz	–	–	–	–	–	–
	HF 64–116 Hz	–	–	–	–	–	–
	BB 0.5–116 Hz	–	–	–	–	–	–

Note. All statistics are reported for data surviving correction for MC either in within-subject OPM but not iEEG (OPM, top panel), within-subject iEEG but not OPM (iEEG, middle panel), and between-method OPM-iEEG, but not within-subject OPM nor iEEG (OPM to iEEG, bottom panel). For each comparison and band, we report the percent of voxels/electrodes surviving correction for MC (% MC), the *M* and *SD* of *z*-scores, and the peak *z*-score with its MNI coordinate and anatomical label. Note, within-subject OPM reliability was computed for 656 virtual electrodes reconstructed at the iEEG electrode locations.

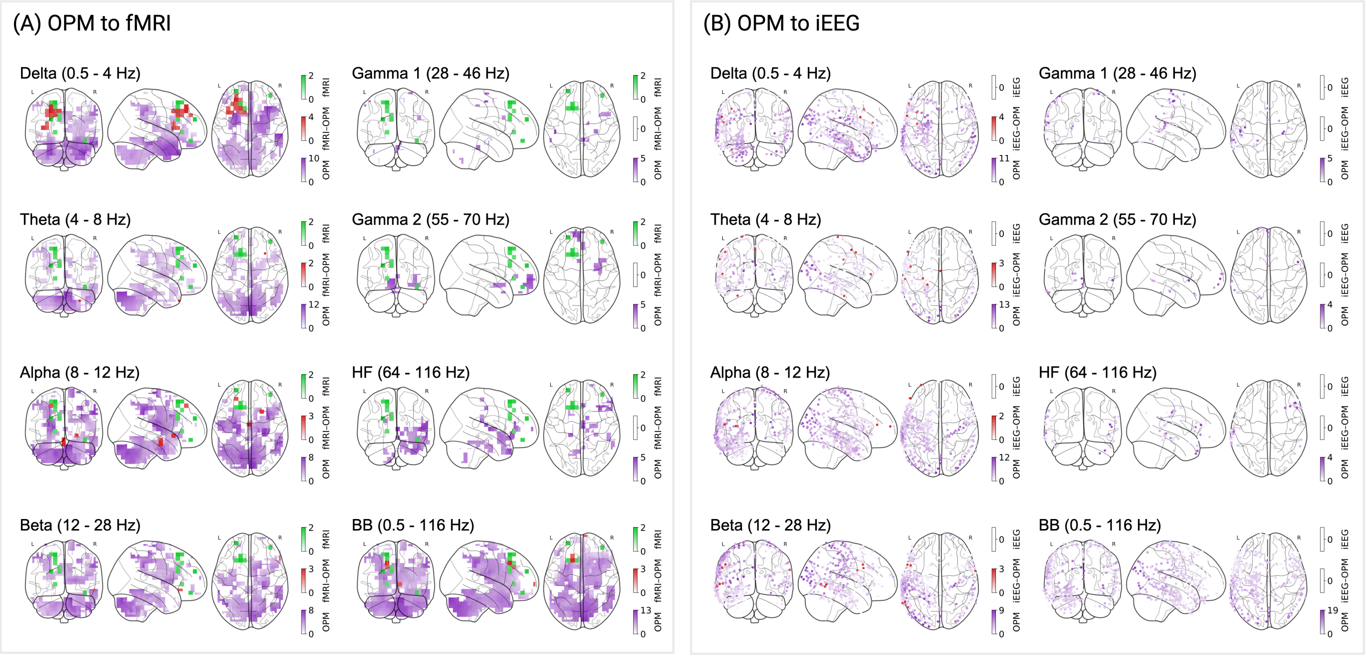

Figure S6: Reliable z-scores exclusive to within-subject and between-method comparisons across OPM, fMRI, and iEEG. Channels/electrodes surviving MC correction for (A) OPM to fMRI: within-subject OPM but not within-subject fMRI (purple), within-subject fMRI but not within-subject OPM (green), and between-method OPM-fMRI but not within-subject OPM nor fMRI (red), and (B) OPM to iEEG: within-subject OPM but not within-subject iEEG (purple), within-subject iEEG but not in within-subject OPM (note, no electrodes survived correction for MC within iEEG only for any frequency), and between-method OPM-iEEG but not in within-subject OPM nor iEEG (red). Color bars reflect *z*-scores (see above).

Table S8: Patterns of reliability exclusive to between-subject OPM, between-subject fMRI, and between-method OPM-fMRI.

					Maxima		
Comparison	Frequency Band	% MC	M_z	SD_z	z	MNI Coord.	AAL Label (Left/Right)
OPM	δ 0.5–4 Hz	64.5%	4.50	1.99	15.18	–32, 24, –32	Temporal Pole Sup (L)
	θ 4–8 Hz	59.4%	4.74	2.32	13.50	8, –80, –40	Cerebellum Crus II (R)
	α 8–12 Hz	56.5%	5.37	2.32	13.59	8, –72, 56	Precuneus (R)
	β 12–28 Hz	69.4%	5.61	2.16	12.52	–32, –80, 40	Occipital Mid (L)
	γ_1 28–46 Hz	9.6%	3.34	0.65	5.55	16, –64, –32	Cerebellum 6 (R)
	γ_2 55–70 Hz	3.5%	3.52	0.62	5.67	16, –48, 16	Precuneus (R)
	HF 64–116 Hz	20.2%	2.98	0.62	5.50	56, 16, 24	Frontal Inf Tri (R)
	BB 0.5–116 Hz	75.0%	8.14	2.99	18.20	–32, –72, 56	Parietal Sup (L)
fMRI	– –	–	–	–	–	–	–
OPM to fMRI	δ 0.5–4 Hz	0.3%	3.52	0.51	4.59	–24, 40, 48	Frontal Sup (L)
	θ 4–8 Hz	0.2%	3.25	0.60	4.43	–32, –8, 56	Precentral (L)
	α 8–12 Hz	0.1%	2.92	0.17	3.16	24, –32, 48	Postcentral (R)
	β 12–28 Hz	0.4%	3.38	0.54	4.23	–48, 16, –24	Temporal Pole Sup (L)
	γ_1 28–46 Hz	–	–	–	–	–	–
	γ_2 55–70 Hz	–	–	–	–	–	–
	HF 64–116 Hz	0.0%	3.48	0.00	3.48	–48, –8, 16	Postcentral (L)
	BB 0.5–116 Hz	–	–	–	–	–	–

Note. All statistics are reported for data surviving correction for MC either in between-subject OPM but not fMRI (OPM, top panel), between-subject fMRI but not OPM (fMRI, middle panel), and between-method OPM-fMRI, but not between-subject OPM nor fMRI (OPM to fMRI, bottom panel). For each comparison and band, we report the percent of voxels surviving correction for MC (% MC), the *M* and *SD* of *z*-scores, and the peak *z*-score with its MNI coordinate and anatomical label.

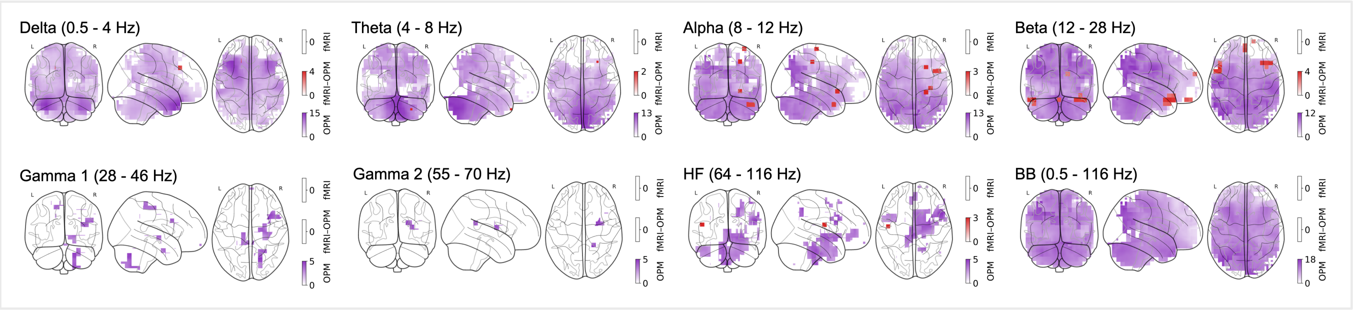

Figure S7: Reliable z-scores exclusive to between-subject and between-method comparisons for OPM and fMRI. Channels surviving MC correction for between-subject OPM but not between-subject fMRI (purple), between-subject fMRI but not between-subject OPM (note, no channels survived correction for MC in between-subject fMRI only), and between-method OPM-fMRI but not between-subject OPM nor fMRI (red). Color bars reflect *z*-scores (see above).
